# Preparation and Characterisation of High-Density Polyethylene/Tannic Acid Composites

**DOI:** 10.3390/polym16233398

**Published:** 2024-12-02

**Authors:** Evangelia Tarani, Myrto Tara, Christina Samiotaki, Alexandra Zamboulis, Konstantinos Chrissafis, Dimitrios N. Bikiaris

**Affiliations:** 1 Laboratory of Advanced Materials and Devices, Department of Physics, Aristotle University of Thessaloniki, GR54124 Thessaloniki, Greece; hrisafis@physics.auth.gr; 2 Laboratory of Chemistry and Technology of Polymers and Dyes, Department of Chemistry, Aristotle University of Thessaloniki, GR54124 Thessaloniki, Greece; myrtotara@chem.auth.gr (M.T.); christinasamiotaki@gmail.com (C.S.); dbic@chem.auth.gr (D.N.B.)

**Keywords:** HDPE, tannic acid, biobased fillers, composites, crystallisation kinetics, thermal stability, antioxidant activity

## Abstract

This research paper highlights the preparation and characterisation of high-density polyethylene (HDPE)/tannic acid (TA) composites, designed to confer antioxidant properties to HDPE, valorising a biobased filler. Indeed, tannic acid is a natural polyphenol, demonstrating, among others, strong antioxidation properties. Using a melt-mixing process, HDPE/TA composites containing various amounts of TA, ranging between 1 and 20 wt%, were prepared, and analyses on their structural, thermal, mechanical, as well as antioxidant properties were conducted. Infrared spectroscopy, differential scanning calorimetry, and X-ray diffraction showed that TA was successfully incorporated into the HDPE matrix. Thermogravimetric analysis evidenced that the onset of thermal degradation decreased, but overall satisfactory stability was observed. The composites exhibited exceptional antioxidant properties, especially the ones with the highest TA content, although it was observed that a high amount of TA had adverse effects on the mechanical performance of the composites.

## 1. Introduction

High-density polyethylene (HDPE) is a thermoplastic synthetic polymer, characterised by limited branching, facilitating regular chain packing during crystallisation, typically resulting in a high crystallinity degree and fast crystallisation kinetics. Its semicrystalline properties contribute to the formation of a dense, rigid yet ductile polymer network, rendering HDPE highly relevant for various technological applications [1]. Besides its relatively high crystallinity, it is a lightweight and non-toxic material, chemically inert [2] and capable of resisting abrasion and corrosion [3]. Furthermore, HDPE exhibits high tensile and bending strength, resulting in remarkable deformability and toughness, and can thus undergo large permanent strains [4]. All these characteristics, along with its affordability and recyclability, make HDPE well suited for numerous applications such as food and beverage containers, shampoo bottles, trash bins, and a multitude of everyday household goods [2]. In addition, HDPE is widely used in the construction sector [1], for instance in pipes [5] and gearing applications [6]. 

Tannic acid (TA), a natural polyphenol, is one of the most abundant natural compounds and, in fact, the fourth after cellulose, hemicellulose, and lignin. It can be extracted from a variety of biomass such as the bark of certain trees (e.g., oak, chestnut, hemlock, and mangrove) or the leaves of certain sumacs as well as plant galls [7]. As a natural polyphenol, it is affordable, widely available, environmentally harmless, and generally biocompatible, while under certain hydrolytic conditions, tannic acid is hydrolysable and biodegradable. Structurally, tannic acid is described as a glucose ester, containing ten galloyl groups, but it is generally found as a mixture of different galloyl esters. These galloyl esters can induce a multitude of interactions, such as hydrogen bonding, π-π interactions, coordination bonding, etc., with numerous functional groups. Tannic acid participates in biological processes through metal chelation, while also exhibiting antioxidant, antibacterial, antiviral, and anti-inflammatory activity [8,9,10,11]. Traditionally, tannic acid was utilised as a tanning agent in leather processing, but it has also found application in the coating and adhesive, pharmaceutical, and food industries. 

Because of their interesting properties, tannic acid and tannins have been combined with a variety of polymers in different applications, the principal ones being as an antioxidant natural filler and as a cross-linking moiety [7,12,13,14,15,16]. Various research groups have investigated tannin as a filler for polylactic acid (PLA). Tannin was found to act as a nucleating agent, while endowing the composites with antioxidant properties, increasing with the filler content, as well as antibacterial properties [17,18,19,20]. Due to their antioxidant activity, tannins have also been investigated as stabilisers for polyolefins such as polypropylene to partially substitute synthetic, oil-based stabilisers [21]. Finally, it has been reported that poly(ε-caprolactone) (PCL) and TA form miscible blends, where the presence of TA slows down the crystallisation of PCL [22].

Natural antioxidants are emerging as promising alternatives to synthetic phenolic compounds, offering antioxidant properties with potentially lower health and environmental risks. However, despite the extensive literature on their use in food products, research focusing on their applications in polyolefins remains limited [23]. Phenolic acids such as gallic acid and chlorogenic acid present limited solubility in non-polar polymer matrices, thus leading to limited effectiveness in stabilising during processing. However, the carboxylic group’s reactivity—which can be utilised in acylation reactions to produce esters with more phenolic hydroxyl groups and enhanced antioxidant activity, solubility, and thermal stability—is their greatest advantage [24]. Lignin, as a phenolic polymer, has also been studied for its antioxidant and stabilising properties in a variety of polymers like polyethylene, PLA, and PCL. In some cases, notably with HDPE, it has been observed that lignin particles form a separate phase, resulting in lower tensile strength [25]. In general, heterogeneity plays a crucial role when it comes to the use of lignin because it decreases mechanical and stabilising properties. Chemically modified lignins have also been investigated, but the modification of the phenolic hydroxyl groups has been shown to compromise their antioxidant properties [26]. Overall, there are still a lot of challenges and knowledge gaps when it comes to the use of natural antioxidants in polymer matrices. In that context, we were interested to explore the potential of tannic acid. 

As mentioned earlier, one of the drawbacks of natural fillers, which are typically hydrophilic, is their lack of compatibility with hydrophobic polymeric matrices, and tannins are no exception [27]. To overcome this issue, chemical modification is frequently employed to improve affinity, notably with esterification and etherification reactions of the hydroxyl groups. For instance, Grigsby et al. studied the modification of tannins using aliphatic anhydrides (acetic, propionic, butyric, and hexanoic anhydrides). The strategy proved successful, and the substitution degree of tannins hydroxyl groups could be controlled; nevertheless, the compatibility of tannins and modified tannins upon incorporation in polypropylene (PP), poly(butylene succinate) (Bionolle), and poly(lactic acid) (PLA) was not particularly examined [28,29,30]. In another approach, Bridson et al. blended condensed tannins with block- or graft-copolymers, such as ethylene vinyl alcohol and maleic anhydride-graft-polyethylene, respectively, before incorporating them in linear low-density polyethylene (final tannin concentration 1% *w*/*w*). According to the authors, this strategy effectively improved tannin compatibility as evidenced by a smaller tannin particle size in the composites [30]. However, the abovementioned strategies require additional processing steps, increasing the complexity and the cost of the composite production. Furthermore, as reported with lignin, the antioxidant activity of tannins can be compromised when its (phenolic) hydroxyl groups undergo chemical modification [31]. 

To the best of our knowledge, the incorporation of tannic acid in HDPE has not been investigated yet, and we were interested to examine whether tannic acid could be added directly into HDPE and if this addition could impart antioxidant properties to the resulting composites. In this context, the present research work focuses on HDPE/tannic acid composites. More precisely, composites containing 1, 5, 10, 15, and 20 wt.% of tannic acid were prepared via melt-mixing, resulting in materials exhibiting a consistent brown appearance, due to the colour of the filler. The composites’ chemical structure was investigated through infrared spectroscopy; differential scanning calorimetry and thermogravimetric analysis were used for the evaluation of the thermal properties, and X-ray diffraction provided data concerning the composites crystallinity. Mechanical behaviour was assessed by tensile testing. Last but not least, antioxidant properties and hydrophilicity were also investigated. Overall, composites with interesting antioxidant properties, appropriate thermal properties, and acceptable mechanical performance were obtained.

## 2. Materials and Methods

HDPE LITEN MB 71 (MFR (190/2.16): 8 g/10 min) was kindly donated by SILON s.r.o. (Sezimovo Ústí-Planá nad Lužnicí, Czech Republic), and tannic acid (TA) (molecular weight 1701.23 g/mol) was supplied by Thermoscientific (Kandel, Germany) in the form of a powder with a dark-yellow appearance. It is an air- and light-sensitive solid, melting at 218 °C.

HDPE composites containing 1, 5, 10, 15, and 20 wt.% of TA were prepared by melt-mixing in a Haake-Buchler reomixer (model 600, Haake-Buchler Instruments Ltd., Saddle Brooke, NJ, USA) equipped with roller blades and a mixing head with a volumetric capacity of 10 cm^3^. Experiments were conducted at 190 °C and 30 rpm for 10 min. Before use, both HDPE and tannic acid were dried overnight under vacuum at 60 °C. The materials prepared by melt-mixing were shaped in films using an Otto Weber Type PW 30 hydraulic press connected with an Omron E5AX Temperature Controller (Grunbach, Germany), at a temperature of 190 ± 5 °C. Hereafter, the materials will be referred to as “TAx”, where x indicates the tannic acid content of the composites, TA0 being neat HDPΕ.

FTIR spectra were recorded either in KBr discs or directly on thin films of the prepared materials, on an FTIR-2000 instrument (Perkin Elmer, Waltham, MA, USA). To prepare the former, each sample was triturated with potassium bromide (KBr), and discs were obtained with a hydraulic press. For the latter, a thin-film fragment was placed on the sampler. Infrared absorbance spectra were collected from 4000 to 450 cm^−1^ using a resolution of 4 cm^−1^ and 32 co-added scans. The presented spectra have been baseline-corrected and -normalised.

The crystallisation and melting behaviour of the HDPE/TA composites were investigated using a Polyma 214 differential scanning calorimeter (DSC) manufactured by NETZSCH, calibrated with indium and zinc standards for accuracy. The samples were precisely weighed to 4 ± 0.2 mg and packed in aluminium pans. A constant nitrogen flow of 40 mL/min was applied during the measurements. To eliminate any thermal history, the samples were subjected to heating from 0 °C to 200 °C (rate 20 °C/min) and held at this temperature for three minutes. Non-isothermal crystallisation scans were conducted at cooling rates of 1, 2.5, 5, and 10 °C/min, and data analysis for assessing non-isothermal crystallisation kinetics was performed using NETZSCH Kinetics Neo software Version 2.6.7.8 (NETZSCH, Selb, Germany) [32]. The DSC curves were further analysed to evaluate the crystallinity fraction of the samples (Equation (1)). This involved determining the ratio of the measured heat of fusion (${∆H}_{m}$) to the theoretical heat of fusion of 100% crystalline material (${∆H}_{m}^{0}$) multiplied by 100%. This ratio, known as the crystallinity fraction ($X_{c}$), takes into account the weight fraction of filler present in the polymer matrix.

(1) $$X_{c}=\frac{{∆H}_{m}}{\left(1-w\right)\;{∆H}_{m}^{0}}\times 100\%$$

where *w* is the weight fraction of HDPE in the composites The theoretical heat of fusion of the 100% crystalline HDPE was taken as 290 J/g [33].

XRD diffractograms were recorded using a MiniFlex II XRD system (Rigaku, Co., Tokyo, Japan) with Cu Kα radiation (0.154 nm) from 5° to 50° (2θ) at a scanning rate of 1°/min. For the analysis, composite films were prepared by compression moulding at 190 °C and then cooled at room temperature. The % crystallinity was calculated from the XRD graphs using Equation (2) [34]:

(2) $$X_{c}=\frac{A_{c}}{A_{c}+A_{m}}\times 100\%$$

where *A_m_* is the area of the amorphous halo and *A_c_* is the area of the crystalline peaks. 

The thermogravimetric analysis (TGA) of the HDPE/TA composites was conducted using a SETARAM SETSYS TG-DTA 16/18 apparatus. Samples, weighting 3 ± 0.5 mg each, were placed in alumina crucibles, while an empty crucible served as a reference. To eliminate the buoyancy effect, a blank measurement was performed, followed by the removal of the experimental curve. The HDPE/TA composites were subjected to heating from ambient temperature to 600 °C at a rate of 20 °C/min, under a nitrogen flow of 50 mL/min. Throughout this study, heat flow, sample mass, temperature, and their first derivatives were continuously recorded. 

Tensile tests were performed using a Shimadzu EZ Test Tensile Tester, Model EZ-LX, with a 2 kN load cell, according to ASTM D882 [35], using a crosshead speed of 5 mm/min. A Wallace cutting press was utilised to cut dumb-bell-shaped tensile test specimens (central portions 5 × 0.5 mm thick, 22 mm gauge length). A minimum of five measurements were performed for each composite, and the results were averaged to obtain the mean values of Young’s modulus, tensile strength at break, elongation at break, and yield stress.

The water contact angle was measured with an Ossila contact angle goniometer L2004A1 (Sheffield, UK) at room temperature (25 °C). To measure the contact angle, a water droplet (5 µL) was gently deposited on the surface of the films of the samples (films prepared via compression moulding). At least three measurements were performed, and the mean value is presented herein.

The morphology of the fractured surfaces of the tensile test specimens was studied with a JEOL JSM 7610F field emission scanning electron microscope (SEM) operating at 5 kV (Tokyo, Japan). To ensure good conductivity for the electron beam, the samples were carbon-coated before observation. The microscope was operated with an accelerating voltage of 20 kV, a probe current of 45 nA, and a counting time of 60 s.

The antioxidant activity of the samples was determined with the 2,2-diphenyl-1-picrylhydrazyl (DPPH) method [36], on thin films of the composites (1 × 1 cm, thickness 5–6 mm). The absorbance of the DPPH solutions was recorded on a UV–Vis spectrometer (UV Probe 1650, Shimadzu, Tokyo, Japan) at 515–517 nm. For the initial set of experiments, each sample film was added to 3 mL of a 0.1 mM DPPH ethanolic solution, while for the second set of experiments, a 0.2 mM DPPH ethanolic solution was employed, and the films of the studied samples were directly immersed in the UV–Vis spectrometer cell.

The absorbance of the DPPH solution (*A_t_*) containing the composite samples was measured at regular time intervals. The free-radical-scavenging activity was calculated according to Equation (3), as follows:

(3) $$Free\;radical\;scavenging\;activity\;\left(\%\right)=\frac{A_{0}-A_{t}}{A_{0}}\times 100\%$$

where *A_0_* is the absorbance of the initial DPPH ethanolic solution used each time (0.1 mM or 0.2 mM).

## 3. Results and Discussion

### 3.1. Preparation and Structural Characteristics

A series of five high-density polyethylene (HDPE)/tannic acid (TA) composites containing 1, 5, 10, 15, and 20 wt% of tannic acid was obtained via melt-mixing as described in the experimental section (190 °C, 30 rpm, 10 min). The composites were dark brown, except for TA1, which exhibited a lighter colour. TA was smoothly incorporated in the HDPE matrix, even in 20 wt%, with no visible signs of agglomeration. 

**Figure 1:**
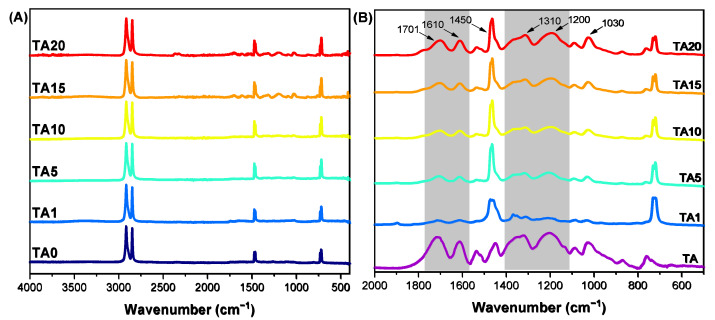
FTIR spectra of the prepared composites. (**A**) Full spectra and (**B**) zoom-in of the 500–2000 cm^−1^ region (TA: tannic acid, TA0: neat HDPE).

The chemical structure of the composites was examined by infrared spectroscopy (Figure 1). In the HDPE (ΤA0) spectrum, the asymmetric and symmetric stretching vibrations of the C-H bonds are observed at 2919 and 2847 cm^−1^, while the CH_2_ bending and rocking deformation vibrations are detected, respectively, at 1472/1461 cm^−1^ and 730/717 cm^−1^ [37]. The peaks arising from the polymer matrix can be detected in the spectra of all the composites (Figure 1A). 

In the spectrum of tannic acid (TA), a broad absorption band is observed at 3500–3070 cm^−1^ (not shown) encompassing the hydroxyl group (O-H) stretching vibrations (free and hydrogen-bonded OH groups) and the C-H bond stretching vibrations of the aliphatic and aromatic -CH_2_- and -CH- groups. In addition, the absorbances at 1701 cm^−1^ and 1610/1450 cm^−1^ are attributed to the stretching vibrations of the C=O and C=C bonds (ester groups and benzene rings in accordance with the presence of aromatic esters in TA). The absorption peaks detected in the 1000–1250 cm^−1^ region can be assigned notably to the C-O bonds stretching and C-OH bonds bending vibrational modes, along with C-C stretching and C-H bending vibrations. Finally, the peak around 1300 cm^−1^ can be attributed to the coupled stretching vibrations of the aromatic C-C bonds and the C=O bonds, and the C-H and C-OH bending vibrations [38,39,40,41]. When it comes to the composites, in TA1, due to the low tannic acid content, signals attributed to TA are not clearly observed. However, when TA content increases over 5%, the characteristic TA signals increase, confirming TA has been successfully incorporated in the HDPE matrix without any alterations (Figure 1B).

### 3.2. Thermal Behaviour and Crystallinity 

The present study investigated, by using DSC and TGA, how the incorporation of TA affects HDPE melting and the crystallisation kinetics of HDPE, as well as its thermal stability. These analyses were complemented by X-ray diffraction (XRD) measurements.

#### 3.2.1. Non-Isothermal Crystallisation Study of HDPE/TA Composites

Crystallisation is a complex phenomenon involving at least two distinct phases: nucleation and growth. In polymers, intricate entropic processes like chain folding and reptation may disrupt this dual fundamental mechanism. Initially, nucleation-controlled mechanisms generally dictate the rate of melt crystallisation; however, as the process progresses, diffusion-controlled processes dominate. Under the notion of melt crystallisation, as temperature rises, the rate of crystallisation usually decreases, showing anti-Arrhenius behaviour. However, despite this intricate character, the Arrhenius law and conventional single-step models, such as the Avrami equation, have been extensively employed to characterise polymer crystallisation dynamics. In this work, the crystallisation of the HDPE/TA composites was studied by DSC, under non-isothermal conditions, with a range of constant cooling rates that were maintained from 1 to 10 °C/min. The “kinetic triplet”, consisting of the activation energy, the pre-exponential factor, and the reaction model, was studied with different DSC scanning rates, as recommended by the ICTAC [32]. 

**Figure 2:**
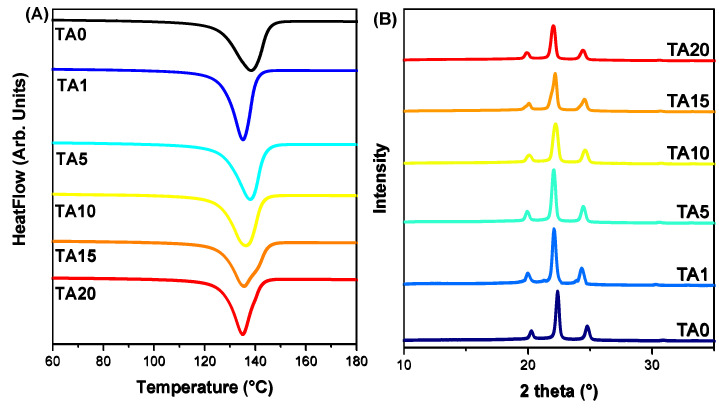
(**A**) Melting peak temperatures of neat HDPE and the HDPE/TA composites, heating rate: 20 °C/min. (**B**) XRD patterns of HDPE/TA composites.

DSC measurements were performed to determine the melting and crystallisation characteristics of pure HDPE and the HDPE/TA composites, as shown in Figure 2A. The peak melting temperature (*T_m_*), the corresponding fusion enthalpy (Δ*H_m_*), and the crystallinity degree (*X_c_*) of all the prepared materials at a heating/cooling rate of 20 °C/min are presented in Table 1. As the filler content increases, the melting temperature of the HDPE/TA composites decreases. On the contrary, the melting enthalpy, and thereby the *X_c_*, in the HDPE/TA composites display an increase, indicating a potential nucleating effect of the TA filler. Neat HDPE exhibits a crystallinity of 71.8%, whereas the TA10 sample shows the highest crystallinity at 82.6%. However, in the case of TA20, lower values are observed, indicating that the higher filler content adversely affects the material properties.

The crystalline structure of the composites was studied by XRD (Figure 2B), and the crystallinity degree was calculated based on Equation (2). XRD analysis verified that the composite materials preserved the typical crystalline structure of HDPE. The degree of crystallinity in the composites with TA at concentrations of 1, 5, 10, 15, and 20 wt.% was found to be 66%, 66%, 62%, 66%, and 59%, respectively. This change in crystallinity, as compared with pure HDPE (76%), is reasonably expected since TA is amorphous [42,43]. The process of TA addition disrupts the organised arrangement of polymer chains in HDPE, which is a common aspect of the incorporation of amorphous fillers into the crystalline polymer matrix. Furthermore, although the inclusion of TA at lower concentrations does result in some enhancement of nucleation, excessive TA content is responsible for a greater disturbance in crystallisation and thus a further lower degree of crystallinity, as observed in the TA20 composite. This observation is in agreement with the literature. For example, Rosli et al. [44] showed that the incorporation of amorphous cellulose in HDPE (2 and 10 wt.%) caused a gradual decrease in the intensity of the (110), (200), and (020) diffraction peaks, indicating a decrease in crystallinity. The same trend was observed by Fei et al. [45], where the addition of 50 wt.% bamboo cellulose diminished the diffraction peak intensity of HDPE, thereby revealing cellulose’s potential to intertwine and disrupt the HDPE crystal structure.

**Figure 3:**
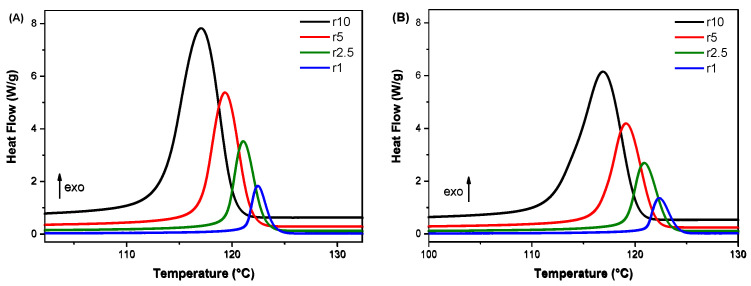
Cooling curves of (**A**) TA1 and (**Β**) TA20 at cooling rates ranging from 1 to 10 °C/min.

Cooling rates from 1 to 10 °C/min were used to study the effect of TA filler on the crystallisation behaviour of the HDPE/TA composites. The cooling curves of two selected composites, TA1 and TA20, are presented in Figure 3. A single, well-defined exothermic peak is observed for all cooling rates. The lowest cooling rates, 1 and 2.5 °C/min, induced rather narrow crystallisation peaks, while the highest cooling rate (10 °C/min), provoked broader crystallisation curves. The peak temperatures shifted to lower temperatures with higher cooling rates, as there is insufficient time to activate nuclei at higher temperatures. The molecular chains exhibit reduced flexibility, and the chain configurations take less time to arrange into more ideal crystallites as they diffuse into the crystallite lattice. Table 2 presents the crystallisation temperature *T_c_* and the crystallisation enthalpy ∆*H_c_* values under non-isothermal crystallisation conditions for the HDPE/TA composites. The *T_c_* values indicate that the addition of TA slightly influences the crystallisation behaviour of HDPE, with variations depending on the cooling rate.

#### 3.2.2. Non-Isothermal Crystallisation Kinetics of HDPE/TA Composites

The kinetic triplet (activation energy, pre-exponential factor, and reaction model) was established using both isoconversional and model-based approaches. Kinetics Neo software from NETZSCH was used to find the kinetic parameters for the melt crystallisation process. Isoconversional methods, including Friedman and Vyazovkin analysis, were used and were further combined with model-based analysis via an n-dimensional nucleation model developed from the Sbirrazzuoli model (SbC) [46,47].

Crystallisation, as a first-order transition, is a process that is characterised by the evolution from an amorphous phase to a crystalline one. The heat that is produced during this process gives a certain degree of crystallinity (X_T_) which is evident from the exothermic peak during analysis [48]. This value is calculated by integrating the heat flow over the temperature range from the start of the crystallisation to the crystallisation point at a certain time, then normalising it by the total heat released during the complete crystallisation. The crystallisation temperature can also be directly related to the crystallisation time, where the temperature at a certain time during crystallisation (T) is connected to the initial crystallisation temperature (T_0_) and the cooling rate (β).

**Figure 4:**
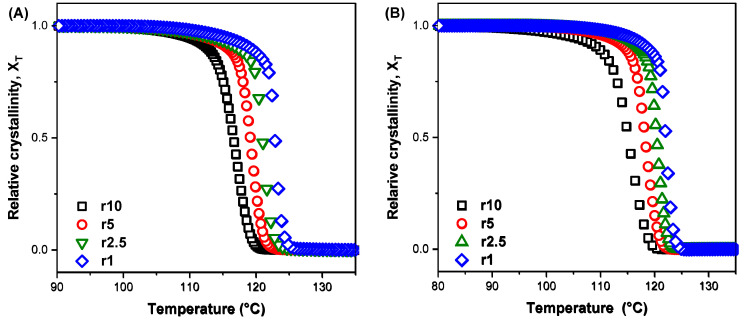
Plots of relative crystallinity versus temperature for non-isothermal crystallisation of (**A**) TA1 and (**Β**) TA5 composites at cooling rates from 1 to 10 °C/min.

Figure 4A,B show the degree of crystallinity (degree of conversion α) as a function of temperature for the non-isothermal melt crystallisation measurements of indicative samples (TA1 and TA5 composites). The curves exhibit a typical sigmoid function; these figures show the important influence the cooling rate has on the nucleation and growth processes. In the last stage of crystallisation, spherulite impingement and crowding cause the upper section of the plot’s curvature to level off. A slower cooling rate extends the time required for crystallisation, as it causes less undercooling, providing more time for the crystallisation process to occur. Table 3 presents the data concerning the half time of crystallisation values, t_1/2_, which is the time the polymer needs to reach 50% of crystallinity, for neat HDPE and the HDPE/TA composites. The consistent t_1/2_ values across different filler contents indicate that TA does not strongly influence the crystallisation rate of HDPE.

#### 3.2.3. Isoconversional Methods

**Figure 5:**
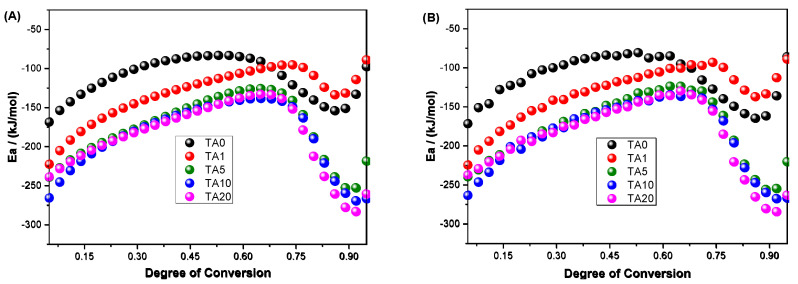
Activation energy (*Eα*) values versus the degree of conversion (α) for the non-isothermal crystallisation kinetics of neat HDPE and the HDPE/TA composites as determined by the (**A**) Friedman method and (**Β**) Vyazovkin analysis.

The various methods of the isoconversional approach brought up by Friedman and the way of studying an entire dataset explained by Vyazovkin were put to use on the activation energies, *Eα*, of the HDPE/TA composites [49,50,51,52]. A plot of the *Eα* values of neat HDPE and the HDPE/TA composites against the degree of conversion α using the mentioned isoconversional techniques is displayed in Figure 5. The results revealed that the activation energy underwent a period of fluctuations during the procedure. This trend indicates that the polymer system experiences more and more difficulty crystallising as the process reaches its end. As the process progresses, the melt crystallisation mechanism changes, and the polymers begin to acquire the required activation energies. However, the *Eα* dependency shows how complicated the melt crystallisation process is: it shows that there are many steps, each with its own energy barriers and contributions. Furthermore, the (E-α) curves computed via the Vyazovkin method (Figure 5B) exhibited a similar trend to those obtained using the Friedman method. The *Eα* values for neat HDPE and the HDPE/TA composites are negative and increase as the degree of conversion increases; this behaviour is attributed to the nucleation control during the process. The HDPE/TA composites present lower activation energy values than those of neat HDPE, indicating that the addition of TA filler accelerates the crystallisation process. 

#### 3.2.4. Model Fitting Methods

Kinetic analysis is based on two major variables: the degree of conversion, α, a fraction indicating complete conversion over some property during processing, and temperature (T). The reaction rate in a single-reaction mechanism is determined by the temperature-dependent reaction rate constant and the degree of conversion-dependent reaction model [48].

The Arrhenius equation usually describes the rate constant, in which *E* (kJ/mol) is the apparent activation energy, *A* (s^−1^) is the pre-exponential factor, *R* (8.314 J/mol·K) is the gas constant, and *T* (K) is the absolute temperature. 

To address the limitations of some models like Avrami’s and Nakamura’s, the Sbirrazzuoli model [53] using Hoffman–Lauritzen theory is often preferred for many polymers. This model considers additional crystallisation processes like lamellar thickening or secondary crystallisation. The Šesták–Berggren (SB) model [54] is an autocatalytic transformation model that includes parameters (*m*, *n*, and *p*) describing the relative contributions of acceleratory and decay regions of the kinetic process. *m* and *n* are the order of autocatalytic reaction and the order of reaction, respectively. The SB (*m*, *n*) model [55,56] has one parameter ratio, *p* = *m*/*n.* The Hoffman–Lauritzen (HL) theory provides insights on primary and secondary nucleation during crystallisation. It assesses the growth rate of crystals on these non-isothermal DSC data using undercooling from the melting point and temperature corrections [46]. 

**Figure 6:**
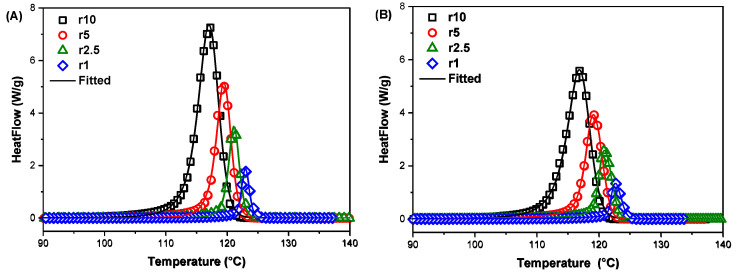
Heat flow curves of (**A**) TA1 and (**Β**) TA20 samples versus temperature and the corresponding fitting of multivariate nonlinear regression of the Sbirrazzuoli model.

The Sbirrazzuolli model was used to compare the rates of crystallisation against temperatures, as shown in Figure 6. Our assumption that the Sbirrazzuolli model takes into account additional crystallisation processes present at higher conversion degrees was confirmed. The results of the HDPE/TA composites are consistent with isoconversional methods and propose a complex melt crystallisation mechanism with multiple steps involving different contributions and activation energies.

According to the results of the Sbirrazzuoli model parameters, identified by optimisation on the melt crystallisation curves for neat HDPE and the HDPE/TA composites, as shown in Table 4, the addition of TA has additively modified the parameters *m* and *n,* exponentially indicating that the TA content slightly influences the crystallisation behaviour of the HDPE matrix. The Kg value was estimated as 0.91 K^2^ and is close to values in the literature [57]. The nucleation parameter Kg of TA1 and TA5 composites was found slightly lower than neat HDPE; indicating that heterogenous nucleation phenomena occurred during the crystallisation of TA1 and TA5. The Kg values suggest that a low amount of TA in the HDPE matrix can facilitate polymer chain nucleation and the initiate crystallisation at higher temperatures during melt crystallisation. Interestingly, the nucleation parameter Kg of TA10 and TA20 composites was higher than that of neat HDPE, indicating that a larger amount of TA filler might hinder nucleation during melt crystallisation. It follows that the higher filler content of the composites restrains the mobility of polymer chains. As a result, the addition of large amounts of TA creates steric hindrances or physical barriers that interfere with the polymer’s ability to nucleate efficiently during the crystallisation process. Furthermore, the pre-exponential factor A of the HDPE/TA composites was higher compared to neat HDPE, as suggested by the calculated activation energy values, and it increased with higher filler content. These results confirm the model’s ability to predict crystallisation kinetics over a large range of cooling rates. Moreover, the model’s ability to accurately predict the onset of crystallisation without requiring isothermal conditions is, in fact, the strongest evidence of its high precision [58].

#### 3.2.5. Thermal Stability 

**Figure 7:**
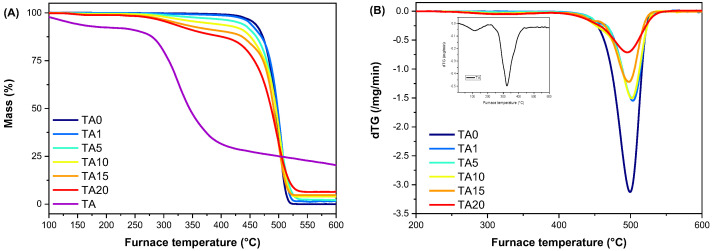
(**A**) TGA thermograms and (**Β**) dTG curves of neat HDPE and the HDPE/TA composites at a heating rate of 20 °C/min under a nitrogen atmosphere.

The thermal degradation of HDPE/TA composites was studied by TGA. Figure 7 presents the TGA thermograms and dTG curves of neat HDPE, TA and TA composites at a heating rate of 20 °C/min under nitrogen atmosphere. 

A weight loss of approximately 8% was noted when tannic acid was heated below 200 °C in a nitrogen environment. This loss is believed to be due to the release of acetic acid and water. The major degradation of tannic acid occurred at approximately 320 °C, an observation consistent with the existing literature [59,60]. When exposed to a nitrogen atmosphere, tannic acid produced a char residue of 19%. 

The TGA curves revealed that both neat HDPE and the HDPE/TA composites exhibit negligible mass loss below 250 °C. The temperatures corresponding to 2.5% (T_2.5_) and 5% (T_5_) mass loss in the HDPE/TA composites, as well as the thermal degradation peak temperature (T_d,max_), are shown in Table 5. Raising the content of tannic acid results in a decrease in thermal stability, as depicted by the decreasing T_2.5_ and T_5_ values of the samples. Neat HDPE shows T_2.5_ and T_5_ values of 438.5 °C and 456.2 °C, respectively. In contrast, ΤA exhibits much lower T_2.5_ and T_5_ values, at 104.3 °C and 135.5 °C, indicating earlier decomposition. As TA content increases in the composites, the T_2.5_ and T_5_ values decrease progressively. For instance, TA1 (1% TA) shows slight reductions in T_2.5_ and T_5_ (431.3 °C and 448.7 °C), while TA20 (20% TA) shows a substantial drop to 273.9 °C and 307.5 °C. However, the T_d,max_ values remain relatively consistent across different composite compositions, indicating that the maximum temperature at which significant degradation occurs is not heavily influenced by the concentration of TA in the HDPE/TA composite. The maximum thermal degradation temperature of TA is observed to be 324 °C, as depicted in Figure 7B (inset). A slight decrease in the maximum decomposition temperature is observed as the TA content increases, with T_d,max_ decreasing from 503.3 °C in TA1 (1% TA) to 495.4 °C in TA20 (20% TA). This suggests that higher amounts of TA may slightly affect the thermal stability of the composite. The residual mass of the composites at 600 °C was found to increase with increasing TA content. 

### 3.3. Mechanical Behaviour

**Figure 8:**
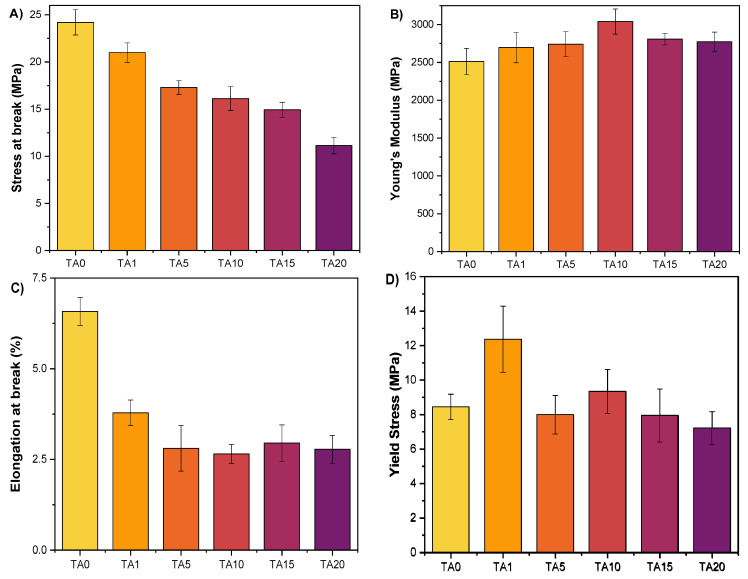
Mechanical properties of neat HDPE and the HDPE/TA composites. (**A**) Stress at break, (**B**) Young’s modulus, (**C**) elongation at break, and (**D**) yield stress.

**Figure 9:**
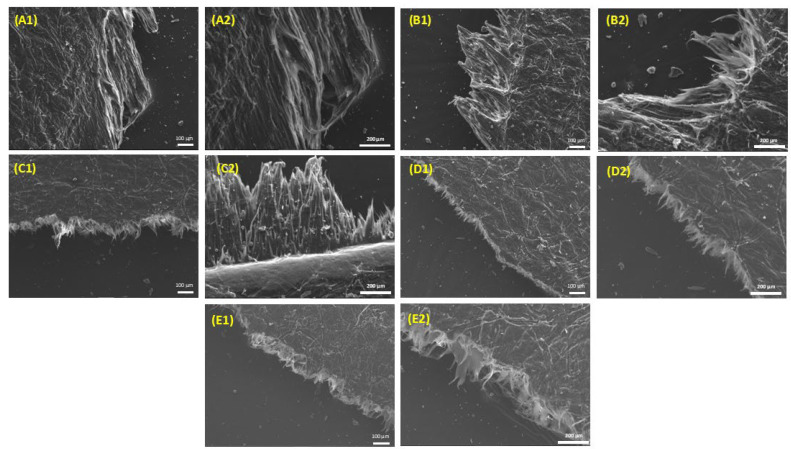
Fractured surfaces after tensile testing as observed by scanning electron microscopy (SEM), at two different magnifications (images labelled with the number “1” were taken at a magnification of ×100; images labelled with the number “2” were taken at a magnification of ×200). (**A**) TA1, (**B**) TA5, (**C**) TA10, (**D**) TA15, and (**E**) TA20.

Mechanical behaviour was assessed by tensile testing (Figure 8 and Table 6). Stress at break (Figure 8A) decreases progressively with the increase in tannic acid. Interestingly, Young’s modulus (Figure 8B) increases initially (up to 10% TA content) and then decreases upon further addition of TA; overall, similar or improved values are observed compared to neat HDPE (TA0). Finally, composites exhibit an elongation at break at around 2.5–3% (Figure 8C), which is considerably lower than that of neat HDPE. These observations, which follow the trend observed in crystallinity with increasing TA content (Table 1), suggest that the composites are stiffer than neat HDPE, which is consistent with the introduction of a rigid filler. These conclusions are further supported by SEM observations of the fractured interfaces (Figure 9). Indeed, as TA content increases, the matrix fibrils at the fracture grow shorter, indicating a more brittle material. This kind of behaviour is typically observed in composites containing natural fillers. 

### 3.4. Hydrophilicity and Antioxidant Properties

**Figure 10:**
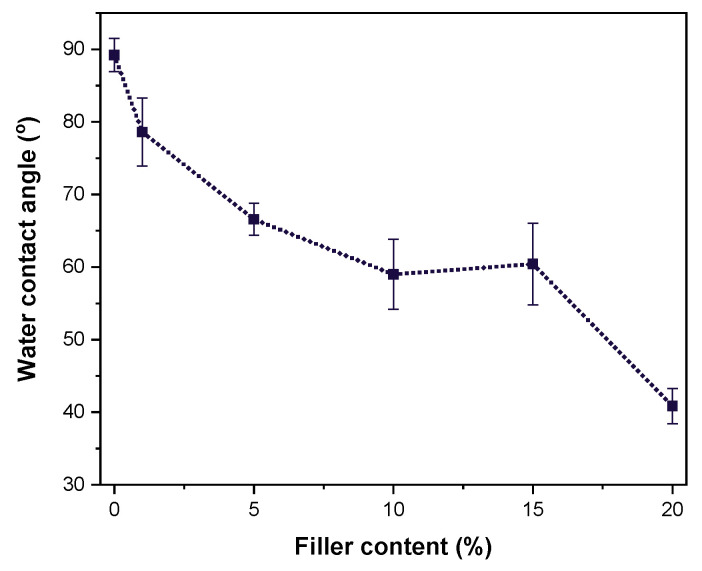
Water contact angle of the composites (the line is drawn to guide the eye).

Water contact angle measurements are presented in Figure 10. As expected, due to the incorporation of tannic acid, which is relatively hydrophilic, a reduction in the water contact angle is observed. TA20 exhibits the lowest value (41°), which is significantly lower than that of neat HDPE [61]. 

**Figure 11:**
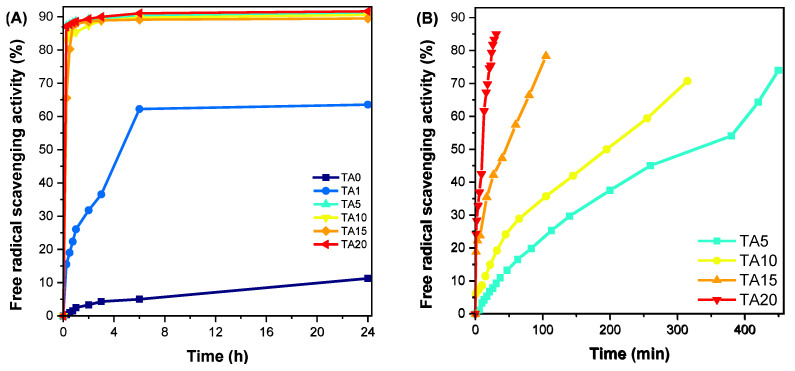
Free-radical-scavenging activity of neat HDPE and the prepared composites with tannic acid. (**A**) All composites and (**B**) composites with 5–20% tannic acid.

Finally, the radical-scavenging ability of the HDPE/TA composites was evaluated with the DPPH method (Figure 11). As expected, neat HDPE exhibited negligible radical-scavenging activity. Tannic acid exhibits high antioxidant activity [62], and its presence conferred important antioxidant properties to the HDPE/TA composites as well. The antioxidant activity is proportional to the TA content, and it is noteworthy that TA20 could decrease the free radical concentration by 85% within 30 min. Even TA1, displaying the lowest activity, could decrease the DPPH radical concentration by 60% within 6 h.

## 4. Conclusions

A series of HDPE composites with commercial tannic acid was prepared to investigate the impact of TA on the behaviour of HDPE. Young’s modulus was slightly improved up to 10% tannic acid content and decreased thereafter, while the other mechanical properties exhibited a decreasing trend with TA content. Crystallisation kinetics were thoroughly studied, evidencing facilitated crystallisation with the incorporation of TA. Although the onset of thermal degradation was shifted to lower temperatures, thermal stability at high temperatures was retained, and overall thermal stability remained satisfactory. Finally, the composites exhibited extremely high radical-scavenging activity. Due to this promising antioxidant potential, UV and thermal ageing studies are in progress to investigate whether the presence of TA could delay oxidative degradation processes. If this hypothesis is confirmed, the present HDPE/TA composites could find applications in the field of active packaging.

## Figures and Tables

**Table 1:** The melting temperature, *T_m_*, enthalpy of fusion, Δ*H_m_*, and degree of crystallinity, *X_c_*, obtained from a heating rate of 20 °C/min, of neat HDPE and the HDPE/TA composites.

Sample	*T_m_* (°C)	Δ*H_m_* (J/g)	*X_c_* (%)
TA0 (Neat HDPE)	138.6	208.2	71.8
TA1	135.2	227.3	79.2
TA5	138.0	224.9	81.6
TA10	136.4	215.7	82.6
TA15	135.6	196.1	75.1
TA20	135.0	182.0	78.4

**Table 2:** The crystallisation temperature, *T_c_*, and the crystallisation enthalpy, ∆*H_c_,* under non-isothermal crystallisation conditions for the HDPE/TA composites (CR: cooling rate).

CR	TA0		TA1		TA5		TA10		TA20	
	*T_c_* (°C)	Δ*H_c_* (J/g)	*T_c_* (°C)	Δ*H_c_* (J/g)	*T_c_* (°C)	Δ*H_c_* (J/g)	*T_c_* (°C)	Δ*H_c_* (J/g)	*T_c_* (°C)	Δ*H_c_* (J/g)
10	116.9	182.7	117.1	229.1	115.5	226.7	116.9	217.0	116.9	185.3
5	119.2	183.4	119.4	230.0	118.4	226.0	119.2	216.8	119.2	185.1
2.5	120.9	183.4	121.1	228.3	120.4	225.8	120.9	215.6	120.9	184.7
1	122.8	181.3	122.5	227.6	122.1	225.1	122.4	215.6	122.4	185.0

**Table 3:** Half time of crystallisation for the HDPE/TA composites.

Cooling Rate (°C/min)	Half Time of Crystallisation, t_1/2_ (min)				
	TA0	TA1	TA5	TA10	TA20
10	107.4	107.3	107.5	107.4	107.4
5	175.3	175.2	175.4	175.2	175.2
2.5	304.8	304.6	304.9	304.7	304.7
1	608.6	608.3	608.7	608.3	608.4

**Table 4:** Parameters of the Sbirrazzuoli model identified by the optimisation of curves of crystallisation from the melt for neat HDPE and the HDPE/TA composites.

Sample	TA0	TA1	TA5	TA10	TA20
*K_g_* (10^5^ Κ^2^)	0.91	0.86	0.90	1.44	1.44
log(*A*) s^−1^	1.93	2.14	2.17	2.69	2.54
*n*	1.59	1.31	1.32	1.56	1.69
*m*	0.38	0.43	0.43	0.51	0.46
*p*	0.28	0.24	0.24	0.26	0.29
*T_m_*	138.3	134.3	138.3	135.8	135.7
*T_g_*	−122.4	−122.2	−122.2	−122.1	−122.0
R^2^	0.99603	0.99848	0.99736	0.99676	0.99829

**Table 5:** TGA results of the HDPE/TA composites. T_2.5_ and T_5_ are the temperatures at which 2.5% and 5% mass loss is observed, respectively. T_d,max_ is the thermal degradation peak temperature.

Sample	T_2.5_ (°C)	T_5_ (°C)	T_d,max_ (°C)
TA0	438.5	456.2	499.4
TA1	431.3	448.7	503.3
TA5	356.4	429.3	501.9
TA10	309.2	366.4	501.5
TA15	282.8	319.7	497.7
TA20	273.9	307.5	495.4

**Table 6:** Mechanical properties of neat HDPE and the HDPE/TA composites.

Sample	Stress at Break (MPa)	Young’s Modulus (MPa)	Elongation at Break (%)	Yield Stress (MPa)
TA0	24.2 ± 1.3	2512 ± 147	6.6 ± 0.4	8.4 ± 0.7
TA1	21.0 ± 1.0	2696 ± 201	3.8 ± 0.3	12.4 ± 1.9
TA5	17.3 ± 0.7	2741 ± 165	2.8 ± 0.6	8.0 ± 1.1
TA10	16.1 ± 1.3	3040 ± 166	2.6 ± 0.3	9.3 ± 1.3
TA15	14.9 ± 0.8	2809 ± 77	2.9 ± 0.5	7.9 ± 1.5
TA20	11.1 ± 0.9	2774 ± 128	2.8 ± 0.4	7.2 ± 0.9

## Data Availability

Data are contained within the article.
